# Comparative analyses of persistence traits in *Escherichia coli* O157:H7 strains belonging to different clades including REPEXH01 and REPEXH02 strains

**DOI:** 10.3389/fmicb.2024.1501956

**Published:** 2024-12-18

**Authors:** Michelle Qiu Carter, Diana Carychao, James L. Bono

**Affiliations:** ^1^Produce Safety and Microbiology Research Unit, U.S. Department of Agriculture, Agricultural Research Service, Western Regional Research Center, Albany, CA, United States; ^2^Meat Safety and Quality Research Unit, U.S. Department of Agriculture, U.S. Meat Animal Research Center, Clay Center, NE, United States

**Keywords:** Shiga toxin-producing *Escherichia coli*, persistence, biofilm, persister cells, VBNC cells, REP strains

## Abstract

Recent application of whole genome sequencing in the investigation of foodborne illness outbreaks has facilitated the identification of Reoccurring, Emerging, or Persistent (REP) bacterial strains that have caused illnesses over extended periods of time. Here, the complete genomes of two *Escherichia coli* O157:H7 (EcO157) outbreak strains belonging to REPEXH01 and REPEXH02, respectively, were sequenced and annotated. Comparative genomics and phenotypic analyses were carried out to identify REP-associated traits. The REPEXH01 strain PNUSAE013245 belonged to clade 8 and shared >96% CDSs with the 2006 spinach-associated outbreak strain EC4115. A 79-Kb genomic island was identified only in PNUSAE013245, and encoded functions related to heavy metals and antibiotic resistances. The REPEXH02 strain PNUSAE043864 belonged to clade 2, like the 2006 Taco John’s restaurants-associated outbreak strain TW14588 and the REPEXH02 strain 2019C-3201. These three strains differed mainly in plasmids and prophages repertoire. Unlike 2019C-3201, PNUSAE043864 carried only the virulence plasmid pO157; unlike TW14588, PNUSAE043864 carried one Stx2a-encoding prophage. Phenotypic assays revealed that all clade 2 strains produced greater amounts of biofilms than the clade 8 strains, although there were no significant differences in adhesion of EcO157 to romaine lettuce. The persistence of EcO157 was further evaluated by quantifying populations of culturable cells, persisters, and Viable But Not Culturable (VBNC) cells for strains inoculated in river water and incubated at 15°C for 14 weeks. The fractions of culturable cells were comparable among the strains tested until 10 weeks post inoculation, when the three clade 2 strains exhibited significantly greater survival than strain PNUSAE013245. The population of persisters for all strains except PNUSAE013245 increased when the incubation time increased and reached up to 24–30% of the total culturable cells. The population of VBNC increased for all strains during the incubation and reached up to 65–80% of the total population. Both persisters and VBNC cells represent the dormancy state of pathogen cells that have increased tolerance to antibiotics and sanitizers. Presence of high portions of persisters and VBNC cells in agricultural environments highlights the need to understand the physiology of bacterial pathogens in leafy greens production environments and the challenges in developing effective control strategies.

## 1 Introduction

Shiga toxin-producing *Escherichia coli* (STEC), primarily the serotype O157:H7 (EcO157), is one of the main bacterial causal agents of foodborne illness outbreaks associated with leafy greens in the United States (Heiman et al., 2015; Marshall et al., 2020). Ruminant animals, primarily cattle, serve as a main reservoir, although STEC strains have been frequently isolated from other animals including feral pigs, wild birds, and wild rodents (Jay et al., 2007; Kilonzo et al., 2013; Navarro-Gonzalez et al., 2020). STEC is released into the environment by fecal shedding and can be further dispersed and disseminated by runoff into watersheds and agricultural fields. Contamination of leafy greens can occur directly or indirectly, via contaminated water, runoff, and even dust containing feces (Brandl, 2006; Berger et al., 2010; Strawn et al., 2013; Cooley et al., 2014). Presence of STEC in the food production environment poses a potential health risk considering that EcO157 can cause severe diseases in humans including hemolytic-uremic syndrome (HUS) at a very low infectious dose (Griffin and Tauxe, 1991; Tuttle et al., 1999).

Natural environments are often considered harsh habitats for enteric pathogens including STEC due to nutrient limitation, fluctuating temperature and humidity, and other stress factors such as predators. STEC has evolved various mechanisms to survive in challenging environments, including formation of biofilms and transition into persisters or a Viable But Not Culturable (VBNC) state. Biofilms are surface-attached microbial communities present in diverse ecological niches (Watnick and Kolter, 2000; Yan and Bassler, 2019). Compared with planktonic cells, biofilm-associated cells are better at coping with environmental stresses and have increased resistance to toxic substances including antibiotics and chemical sanitizers. Biofilms of STEC were reported on plants and on the surfaces of material commonly used by food industries, and under the conditions relevant to fresh produce production (Ryu and Beuchat, 2005; Yaron and Romling, 2014; Carter et al., 2016; Carter et al., 2019). Biofilms of STEC contributed to their environmental persistence and were associated with an increased risk of STEC contamination in preharvest and food processing environments (Vogeleer et al., 2014; Carrascosa et al., 2021; Chitlapilly Dass and Wang, 2022).

Bacterial persisters refer to cells in a transient dormant-like state where metabolism is slowed and growth is arrested (Balaban et al., 2004; Kint et al., 2012). As a result, persisters can survive challenges with antibiotics better than the bulk of the population (Balaban et al., 2004; Lewis, 2010). Unlike antibiotic-resistant mutants, persisters can revert to a metabolically active state and resume growth when the inducing stress is removed. Although persister cells are thought to arise stochastically, studies have suggested that production of persisters can be genetically controlled and induced in response to antibiotics and environmental stresses (Dorr et al., 2009; Nguyen et al., 2011; Lewis, 2012; Wu et al., 2012; Johnson and Levin, 2013). Consistently, a large subpopulation of persister cells was detected for EcO157 when cells were in the stationary growth phase or in the field water when the total population started to decline (Thao et al., 2019). Persisters of EcO157 were also detected on the inoculated lettuce plants and the level of persisters appeared to be associated with the low water availability on the lettuce leaves, a common condition for lettuce grown in the field (Munther et al., 2020).

Bacterial VBNC cells are those that remain viable but are unable to grow on routine culture media (Xu et al., 1982). A VBNC state can be induced by numerous chemical and physical factors, including nutrient starvation, abnormal temperatures, osmotic stress, heavy metals, and even exposure to white light (Oliver, 2010). Induction of VBNC state is thought to be a common adaptive mechanism that allows bacteria to survive in challenging environments. Compared to culturable cells, cells in VBNC exhibit a marked difference in cellular morphology, membrane composition, metabolism, and physical and chemical resistances. Like persisters, VBNC cells are highly tolerant to antibiotics and other stress challenges and are thought to arise stochastically and can be induced under similar conditions that induce persisters (Ayrapetyan et al., 2015; Ayrapetyan et al., 2018). Transition of STEC cells into VBNC was reported in response to various environmental stresses and under food processing and preservation conditions (Li et al., 2014). VBNC cells of EcO157 were detected in environmental samples and on lettuce leaves (Wang and Doyle, 1998; Dinu and Bach, 2011). Conditions that were reported to induce a VBNC state in EcO157 included low temperature, UV disinfection, and the common sanitizers used in the fresh produce industry (Zhang et al., 2015; Wei and Zhao, 2018; Truchado et al., 2021).

Recent application of whole genome sequencing in the investigation of foodborne illness outbreaks has facilitated the identification of Reoccurring, Emerging, or Persistent (REP) bacterial strains that have caused illnesses over extended periods of time. During March–August of 2018, a large, multistate outbreak of EcO157 infection associated with consumption of romaine lettuce occurred in the U.S. and Canada, resulting in 240 people from 37 states being infected (Bottichio et al., 2020). An epidemiological and traceback investigation revealed that romaine lettuce grown in Yuma, Arizona, was the source of the outbreak while irrigation canal water was likely the source of contamination. This outbreak EcO157 strain was found to be a persistent strain belonging to REPEXH01 that was first reported to PulseNet in 2017. Furthermore, the REPEXH01 strains had been linked to 14 different outbreaks in the U.S. from 2017 to 2022.^1^ The REPEXH02 strain is a reoccurring EcO157 strain that has been linked to multiple leafy greens-associated outbreaks from 2016 to 2019 in the U.S., including the two large multistate outbreaks associated with consumption of romaine lettuce during October–December of 2018 and September–December of 2019 (Waltenburg et al., 2021; Chen et al., 2023). In both outbreaks, sources were traced to California growing regions, including Santa Maria in 2018 and the Salinas Valley in 2019. Presence of EcO157 REP strains in leafy greens-growing regions poses challenges to the development of effective mitigation strategies. Currently, little is known about genetic loci, molecular factors, and phenotypic traits underlying the persistence and reoccurrence of the REP strains. REPEXH01 strains belong to clade 8, like the strains linked to the 2006 spinach-associated outbreak in the U.S. The clade 8 strains were reported to be associated with more severe diseases, as defined by higher HUS and hospitalization frequencies (Manning et al., 2008); REPEXH02 strains belong to clade 2, a predominant linage which consists of a group of genetically diverse strains. In this study, we assembled a group of EcO157 outbreak strains belonging to different clades, including REPEXH01 and REPEXH02 strains, to assess genetic loci and phenotypic traits contributing to environmental persistence of EcO157.

## 2 Materials and methods

### 2.1 Bacterial strains, reagents, and growth media

All *E. coli* strains used in this study are listed in Table 1. The strains were routinely maintained and cultured in Luria-Bertani half-salt (5 g NaCl/liter) (LBHS). Ciprofloxacin (Sigma-Aldrich) was prepared according to the manufacturer’s instructions.

**Table 1:** Strains used in this study.

Strains^a^	Associated outbreaks/location and year	Clade^b^	SNP cluster^c^	*stx* genes	Genomic features (GenBank Accession #)			References
					Chromosome (bp)	Virulence plasmid pO157 (bp)	Other plasmids (bp)	
PNUSAE013245	Romaine lettuce/U.S., 2018	8	PDS000181369.83	*stx*_2a_ + *stx*_2c_	5,557,287 (CP126906.1)	94,623 (CP126907.1)	None	This study
EC4115 (RM6069)	Spinach/U.S., 2006	8	PDS000181369.83	*stx*_2a_ + *stx*_2c_	5,572,075 (CP001164.1)	94,644 (CP001163.1)	37,452 (CP001165.1)	Eppinger et al. (2011)
PNUSAE043864	Romaine lettuce/U.S., 2019	2	PDS000035073.188	*stx*_2a_	5,450,859 (CP126904.1)	92,750 (CP126905.1)	None	This study
PNUSAE020169(2019C-3201)	Romaine lettuce, multiple outbreaks/U.S., 2016–2019	2	PDS000035073.188	*stx*_2a_	5,488,442 (CP090856.1)	92,724 (CP090859.1)	87,920 (CP090857.1); 61,933 (CP090858.1)	Chen et al. (2023)
TW14588	Taco John (Iceberg lettuce)/U.S., 2006	2	PDS000035067.11	*stx*_1a_ + *stx*_2a_ + *stx*_2a_	5,578,816 (CM000662.1)	92,381 (DS999999.1)	None	Eppinger et al. (2011)
EDL933	Ground beef/U.S., 1982	3	PDS000004368.90	*stx*_1a_ + *stx*_2a_	5,528,445 (AE005174.2)	92,077 (AF074613.1)	None	Burland et al. (1998), Perna et al. (2001)

a Like strain EC4115, strain RM6069 is a clinical isolate linked to the 2006 spinach-associated outbreak in U.S. and used in the phenotypic assays described in this study.

b Clade was determined *in silico* using the SNP profiles described previously (Riordan et al., 2008).

c The SNP cluster was identified using the “*E. coli* and Shigella” database in Pathogen Detection available at NCBI website.

### 2.2 Genome sequencing, annotation, and analyses

Genomes of two EcO157 outbreak strains were sequenced on a PacBio Sequel IIe system as described previously (Carter et al., 2023). Briefly, bacterial DNA was extracted from exponential phase cultures grown in LB broth using Qiagen Genomic-tip 100/G columns (Valencia, CA). Purified genomic DNA (10 μg) was sheared to a 30 Kb target fragment length using g-TUBEs (Covaris, Woburn, MA) and concentrated with 0.45x volume AMPure PB beads (Pacific Biosciences). Five μg sheared DNA was used to make PacBio sequencing libraries using the SMRTbell Prep Kit 3.0 according to the manufacturer’s protocol and barcoded using the SMRTbell barcoded adapter plated 3.0. The Sequel II binding kit 3.2 and Sequel II sequencing plate 2.0 were used to run the library with the application HiFi reads and a 30-h movie time with a 6-h pre-extension. PacBio reads were assembled using Microbial Genome Analysis in SMRT analysis v 10.1 and contigs imported into Geneious Prime^®^ (Dotmatics). The overlapping sequence on the ends of the contigs were removed from the 5′ and 3′ ends to generate circularized chromosomes and plasmids. Closed chromosomes were reoriented using Ori-Finder 2 (Luo et al., 2014) and both genomes were oriented to the same start position. The closed chromosome and plasmids were manually polished by mapping Illumina and PacBio reads to the chromosome and known plasmids using Geneious mapper. Unused reads were *de novo* assembled using the Geneious assembler for small plasmid identification. All genomes and plasmids were annotated with the NCBI Prokaryotic Genome Annotation Pipeline (Tatusova et al., 2016). The GenBank accession numbers are listed in Table 1. The complete genome sequences were submitted to PHASTER (Arndt et al., 2016) for identification of prophage and prophage-like elements. Clade association was determined based on the SNP profile reported previously (Riordan et al., 2008).

### 2.3 Curli production and biofilm formation

Curli fimbriae were examined by growing each strain at 26°C for 48 h on the Congo Red indicator (CRI) plates, consisting of LB agar plates without sodium chloride and supplemented with 40 μg/mL of Congo Red dye and 10 μg/mL of Coomassie Brilliant Blue, as described previously (Carter et al., 2011). Curli-producing strains were indicated by red colonies, whereas curli-deficient strains were indicated by white colonies on CRI plates. Biofilm assays were carried out as described previously (Carter et al., 2016; Carter et al., 2018). Briefly, 1 ml of Luria-Bertani no-salt (LBNS) broth inoculated with 1 × 10^6^ cells/ml was aliquoted into a borosilicate glass tube and then incubated statically at 26°C for 48 and 120 h. At the end of each incubation, the planktonic cells were removed carefully, and the tubes were rinsed twice with 1 ml sterile distilled water and then stained with 1 ml 0.1% crystal violet at room temperature for 30 min. The dye was then removed gently, and the tubes were washed twice with sterile distilled water. The crystal violet bound to the glass tube was solubilized in 0.5 mL of 33% acetic acid and the absorbance was determined at 570 nm using a BioTek Synergy HT microplate reader (Agilent, Santa Clara, CA). Tubes with uninoculated media served as negative controls. Each data set was the average of results from at least three biological replicates. The differences in attached biomass, represented by the absorbance at 570 nm, among the strains were assessed by the adjusted *p*-value of the Tukey’s multiple comparisons test after a One-way ANOVA test (*p* ≤ 0.05).

### 2.4 Lettuce attachment assay

Single colonies of each EcO157 strain grown in LBNS medium at 28°C for 18 h were used to prepare cell suspensions in potassium phosphate buffer (10 mM, pH 7.0) (KP buffer) at a concentration of 0.05 OD_600_ for lettuce inoculation. The actual concentration of each cell suspension was determined by plate count on CHROMagar™ O157 (DRG International, Springfield, NJ) agar plates. Organic romaine lettuce heads were purchased from a local retail store. On the day of experiment, lettuce disks were cut with a 2 cm cork borer from the middle of each leaf on both sides of the main vein. Four disks were taken from each leaf, and each disk was inoculated with one biological replicate. A total of eight biological replicates were examined for each strain. For plate count, two technical replicates were plated out for each sample. EcO157 cells were spot inoculated onto each disk with 10 spots on each disk, 5.0 μL of cell suspension per spot, at the outside face of the lettuce leaf. The inoculated disk was placed in a covered petri dish and incubated at 25°C for 1 h. At the end of the incubation, each disk was transferred into a 50 mL conical tube containing 10 mL KP buffer. The tube was inverted gently five times to wash off unattached cells. The disk was transferred to a new tube containing 10 mL KP buffer. The EcO157 cells attached to the disk were released into 10 mL KP buffer by vortexing the tube on a Vortex (Scientific Industries Vortex Genie 2 with a 3-inch platform) at 3200 RPM for 1 min. The released cells, termed as “loosely attached cells,” were quantified by plate count on CHROMagar™ O157 agar plates. The same disk was then transferred into a new tube containing 10 mL KP buffer. The tube was then sonicated in an Ultrasonic Cleaner Water Bath (VWR Scientific Aquasonic 75 T) at full strength for 1 min to release cells that were tightly attached to the disk, which was termed as “tightly attached cells.” The released cells were quantified by plate count on CHROMagar™ O157 agar plates.

### 2.5 Quantification of *Escherichia coli* O157:H7 persisters

The assay for persister enumeration was carried out as described previously using the Ciprofloxacin-based lysing method with slightly modification (Thao et al., 2019). The minimal inhibitory concentration (MIC) for ciprofloxacin for each EcO157 strain was determined using the broth microdilution method (Wiegand et al., 2008), except that LBHS broth medium was used. River water was collected from the Salinas River, California (Location: 36°7′0.32″N, 121°1′41.57″W) in April 2023. About two liters of river water was autoclaved twice, each at 121°C for 1 h, and stored at 4°C for the following experiments. Total carbon, organic carbon, and nitrogen in river water was 47.5 mg/L, 4.0 mg/L, and 1.03 mg/L, respectively, and pH at 15°C was 8.47.

Overnight cultures of EcO157 strains grown in LBHS medium were used to inoculate the sterile river water. Three biological replicates were examined for each strain and two technical replicates were plated out per sample for plate count. Briefly, cells of each culture were collected by centrifugation at 8,000 g for 3 min and resuspended in 50 mL sterile river water at an OD_600_ of 0.5. The actual inoculated *E. coli* O157:H7 cells were determined by plate enumeration. The inoculated river water was incubated at 15°C statically for 14 weeks (98 days). The population of persisters in each river water culture was determined weekly for 6 weeks then sampled monthly for an additional 2 months. On the day of sampling, the total population of *E. coli* O157:H7 was determined by plate count. An aliquot of 2 ml river water culture was transferred to a new culture tube and ciprofloxacin was added at a concentration of 10 × MIC. Each culture tube was returned to 37°C and incubated with gentle shaking for 24 h. At the end of incubation, each culture was first centrifuged at 8,000 × g for 3 min, washed once with an equal volume of KP buffer, and then resuspended in an equal volume of KP buffer for plate count. The CFUs on LBHS agar plates represent the drug tolerant persisters that survived the ciprofloxacin challenge.

### 2.6 Quantification of *Escherichia coli* O157:H7 VBNC cells

The abundance of VBNC cells in the river water was assessed for all cultures that were sampled for persister quantification using viability PCR (vPCR) described previously (Zhao et al., 2021) with minor modifications. Briefly, on the day of sampling, an aliquot of 5 mL river water culture was transferred to a 5 mL centrifugation tube. The EcO157 cells were collected by centrifugation at 10,000 g for 3 min and the cell pellet was resuspended in 400 μL of river water supernatant. The resuspended cells were then treated with 25 μM PMAxx^™^ (Biotium Inc., Fremont, CA) in the presence of PMA Enhancer for Gram Negative Bacteria (Biotium Inc) in 500 μL total volume in the dark for 10 min followed by exposing the cell suspension to light for 15 min in a PMA-Lite^™^ 2.0 LET Photolysis Device (Biotium Inc.). The treated cells were then collected by centrifugation at 8,000 g for 3 min and the cell pellet was used for genomic DNA (gDNA) extraction using a Qiagen DNeasy PowerWater extraction kit. The purified gDNA was eluted in 100 μL EB buffer and stored in −20°C for digital PCR. The digital PCR was performed in a 12-μl volume containing 1x Qiacuity EvaGreen PCR mastermix (Qiagen Inc), 1 μL of 100-fold diluted genomic DNA, 0.4 μM of forward (5′-GCACTAAAAGCTTGGAGCAGTTC-3′) and reverse primer (5′-AACAATGGGTCAGCGGTAAGGCTA-3′) that are specific to *E. coli* O157:H7 (Biotium Inc.), and 0.25 U restriction enzyme EcoRI-HF (New England Biolabs, Ipswich, MA). DNA samples were digested by EcoRI-HF to improve random template partitioning during dPCR. The PCR reaction mixture was first incubated at room temperature for 15 min, then loaded to a QIAcuity Nanoplate (8.5 k 24-well) (Qiagen Inc.). Thermal cycling was run on a QiAcuity Eight (Qiagen Inc.) with 40 cycles of 15 s at 95°C, 30 s at 52°C, and 15 s at 72°C, and a final incubation at 40°C for 5 min. The data were analyzed using the QiAcuity Software Suite. Three biological replicates were examined for each strain and vPCR was run in duplicates for each sample. The VBNC population is the difference between the total population derived from digital PCR and the culturable population derived from the plate counts.

## 3 Results

### 3.1 Genome signature of *Escherichia coli* O157:H7 REPEXH01 strain PNUSAE013245

**Figure 1:**
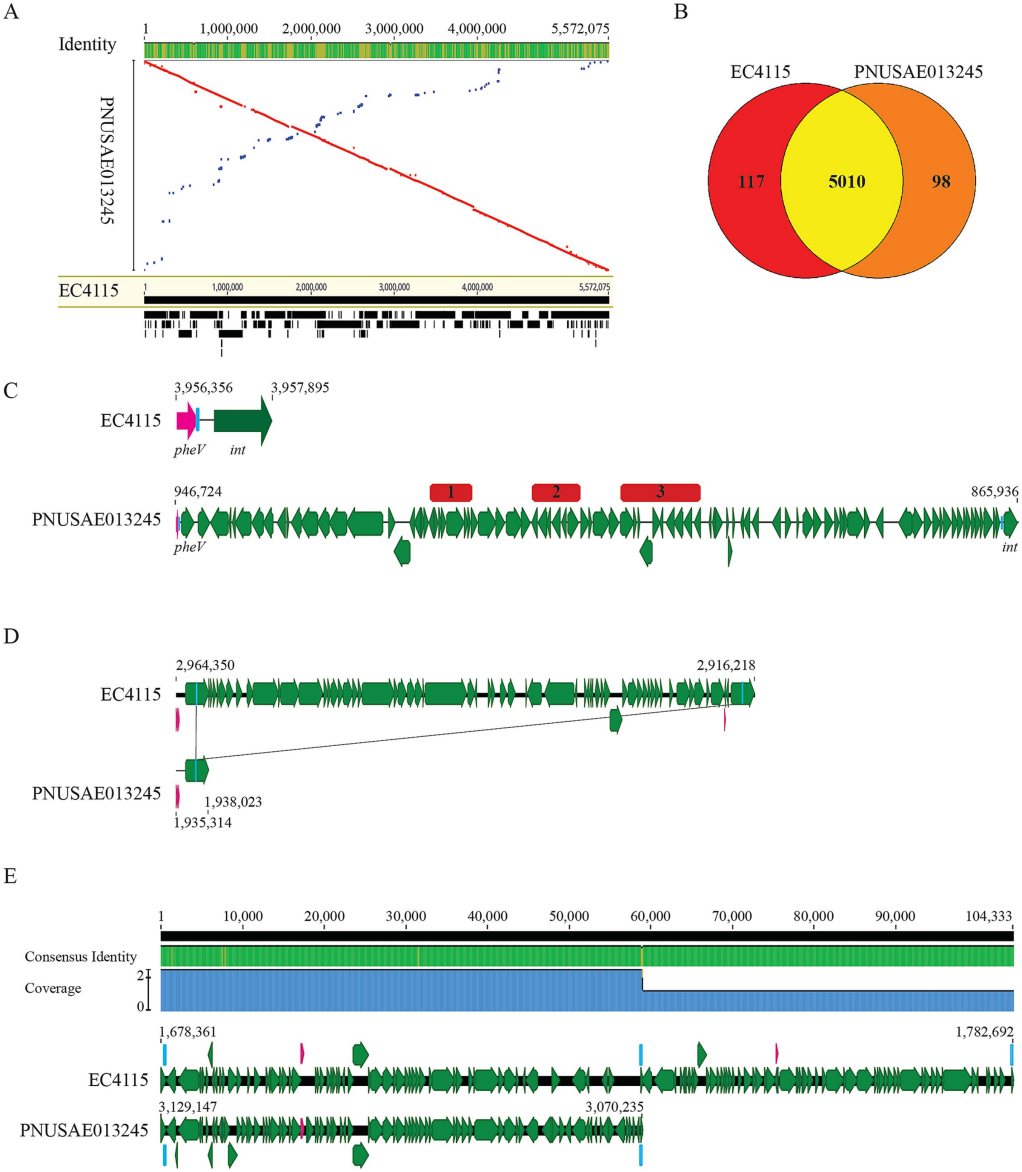
Genomic features of the REPEHX01 strain PNUSAE013245. **(A)** LASTZ alignments of chromosomes of the REPEXH01 strain PNUSAE013245 and the 2006 spinach-associated outbreak strain EC4115. The numbers indicate the aligned base pair position and the black blocks and lines at the bottom represent the 358 target DNA sequences used in the pairwise alignment. The alignment was performed in Geneious Primer^®^2024.0.5 using LASTZ (Version 1.04.15) with the following parameters: Step Length: 20; Seed Pattern: 12 of 19; and HSP Threshold Score (upper limit): 3,000. **(B)** Venn Diagram of shared and strain specific CDSs between the two genomes. Numbers of shared and strain specific orthologs were calculated in EDGAR 3.2 using default parameters. **(C)** Chromosomal location of the large insertion in the REPEXH01 strain PNUSAE013245 and the corresponding chromosomal position in strain EC4115. Pink arrows represent tRNA genes; Blue blocks within the tRNA gene *pheV* represent the putative integration site (5′-ATTCCGAGTCCGGGCACCA-3′). An identical integration sequence was identified on the right border of the large insertion in the REPEXH01 strain PNUSAE013245. Red blocks represent mercury resistance genes (1), integron encoded AMR genes (2), and other AR genes (3). **(D)** Chromosomal position of the 45-Kb genomic island (GI) present in strain EC4115 but absent in the REPEXH01 strain PNUSAE013245. Deletion of this GI in strain PNUSAE013245 was likely to be mediated by the recombination between the two DRs (blue blocks) within the border genes (Locus_tags: ECH74115_3147 and ECH74115_3214), both encoding a conserved hypothetical protein and share 98.3% identity in their coding sequences. Pink arrows represent tRNA genes. **(E)** Sequence comparison of the chromosomal regions containing a large deletion in strain PNUSAE013245. Blue blocks represent DRs while pink arrows represent tRNA genes. In strain EC4115, three DRs are located within an intact *ompW* gene (Locus_tag, ECH74115_1743; 639 bp) and two truncated *ompW* genes (Locus tags, ECH74115_1820 and ECH74115_1887; 117 bp). In strain PNUSAE013245, two DRs are located within an intact *ompW* gene (Locus_tag, DDA95_16160; 639 bp) and a truncated *ompW* gene (Locus_tag, DDA95_15755; 117 bp).

Strain PNUSAE013245 is a clinical isolate linked to the large multistate outbreak associated with romaine lettuce in the spring of 2018 in the U.S. and Canada (Bottichio et al., 2020). The genome of strain PNUSAE013245 is composed of a 5,557,287 bp chromosome and a large plasmid, pO157 (94,623 bp) (Table 1). PHASTER analysis revealed a total of 24 prophage and prophage-like elements on the chromosome, of which, 13 were intact, including a Stx2a-prophage and a Stx2c-prophage (Table 2; Supplementary Table S1). Clade lineage analyses revealed that strain PNUSAE013245 belonged to clade 8, like the EcO157 strains linked to the 2006 spinach-associated multistate outbreak in the U.S. Pairwise genome alignment of PNUSAE013245 with the spinach-associated outbreak strain EC4115 revealed that the two chromosomes shared a great number of syntenic regions (Figure 1A). The virulence plasmid pO157s in the two strains were nearly identical (Data not shown). Consistently, nearly 96% of total annotated CDSs were present in both strains (Figure 1B). Variations between the two genomes were mainly attributed to three chromosomal regions and the second plasmid harbored only by strain EC4115, which mainly encoded conjugation function (Table 1). Note that this small plasmid was not reported in genomes of other 2006 spinach-associated outbreak strains including TW14359 (BioSample number: SAMN02604255). In strain PNUSAE013245, a 79-Kb genomic island (GI) was identified immediately downstream of the tRNA gene *pheV*, which carried genes related to heavy metal resistance, such as mercury resistance genes *merR* and *merTPCADE*, and antibiotic resistance genes, such as *sul1*, *qacE*, *aadA1*, and *dfrA1* that are often found on integrons, and *aph(3′)-Ib*, *aph (6)-Id*, *floR*, *sul2*, *tetR*, and *tetA* (Figure 1C; Supplementary Table S2). This genomic site remains unoccupied in strain EC4115 (Figure 1C). BLASTn search of all genes located on this GI against the EC4115 genome identified homologs of several genes related to mobile genetic elements (MGEs) but failed to reveal any homologs of the heavy metal resistance genes or AR genes. A homolog of the complete GI (79 Kb) was not detected in any of the other EcO157 strains examined in this study. A search of the NCBI nr database by Megablast revealed homologs of a complete GI in strains PNUSAE013304 that was isolated by CDC (GenBank accession number, CP034936.1) and belonged to the same SNP cluster as the strain PNUSAE013245, and Z1323MEC0002 that was isolated in South Korea (GenBank accession number, CP148314.1). The GIs in both strains are nearly identical to the GI in strain PNUSAE013245 (% Identity, 99.98). A partial GI (26–30 Kb) that carries the mercury resistance genes (*merR*, *merTPCADE*) was found in a *Salmonella enterica* Newport strain (GenBank accession number, CP074217.1) and in a clinical enterotoxigenic *Escherichia coli* (ETEC) strain that was isolated in Saudi Arabia (GenBank accession number, CP035826.1).

**Table 2:** Genomic characteristics and chromosomal positions of Stx-prophages in EcO157 strains.

Strains	Clade	SNP cluster	Stx-prophages^a^				
			*stx* genes	Chromosomal locations	Size (bp)	%GC	CDS
PNUSAE013245	8	PDS000181369.83	*stx*_2a_	*argW* - (1,605,930–1,664,899) - *yfdC*	58,970	49.2	84
			*stx*_2c_	sbcB* - (2,134,108–2,191,309) - sbcB*	57,202	51.0	81
EC4115	8	PDS000181369.83	*stx*_2a_	*argW* - (3,297,022–3,234,765) - *yfdC*	62,258	49.3	79
			*stx*_2c_	sbcB* - (2,720,133–2,662,926) - sbcB*	57,208	51.0	70
PNUSAE043864	2	PDS000035073.188	*stx*_2a_	wrbA* - (3,424,922–3,360,914) - wrbA*	64,009	50.0	92
PNUSAE020169 (2019C-3201)	2	PDS000035073.188	*stx*_2a_	wrbA* - (1,032,480–1,095,175) - wrbA*	62,696	49.9	91
TW14588	2	PDS000035067.11	*stx*_1a_	btsS mlrA* - (3,889,622–3,941,374) - mlrA*	51,753	52.4	73
			*stx*_2a_	*argW* - (3,579,351–3,640,522) *- yfdC*	61,172	50.4	81
			*stx*_2a_	wrbA* - (5,388–1; 5,578,816–5,522,473) - wrbA*	61,732	50.0	82
EDL933	3	PDS000004368.90	*stx*_1a_	btsS mlrA* - (2,966,157–3,015,072) - mlrA*	48,916	52.0	64
			*stx*_2a_	wrbA* - (1,330,836–1,392,491) - wrbA*	61,663	49.4	69

a Prophages were initially identified by PHASTER and manually corrected by mapping the corresponding bordering regions in the genome of K-12 sub-strain MG1655. Genes represent the ones flanking the prophage genome. *truncated genes.

Furthermore, two large deletions were identified in the chromosome of strain PNUSAE013245 when compared with the chromosome of strain EC4115. The first one was about 45 Kb and located within a prophage genome in strain EC4115 (Chromosomal location: 2,880,663–2,999,662) (Figure 1D). Deletion of this GI in REPEXH01 strain PNUSAE013245 was likely to be mediated by the recombination between the two DRs (5′-AAACAAAACGGAAAA-3′) within the two border genes (Locus_tags: ECH74115_3147 and ECH74115_3214), both encoding a conserved hypothetical protein that shared 98.3% identity in their coding sequences. Genes within this 45-Kb GI were mainly involved in biosynthesis of phage structural proteins. The second deletion was also about 45 Kb and belonged to a prophage genome in strain EC4115 (Chromosome location: 1,678,881–1,782,574) (Figure 1E). Deletion of this GI in strain PNUSAE013245 was likely to be mediated by the recombination between the two DRs (5′- CAGTGTGGTACATGGATATCGATACCAC -3′) within the two truncated *ompW* genes. Most genes on this GI encoded phage structural proteins.

The Stx2a-prophage in strain PNUSAE013245 exhibited high sequence similarity (~93.3% sequence identity) with the Stx2a-prophage in strain EC4115 and inserted adjacent to the tRNA gene *argW*, like the Stx2a-prophage in strain EC4115 (Table 2); Similarly, the Stx2c-prophage in strain PNUSAE013245 was nearly identical to the Stx2c-prophage in strain EC4115 (~99.9% sequence identity) and inserted within the gene *sbcB*, like the Stx2c-prophage in strain EC4115 (Table 2).

### 3.2 Genome signature of STEC O157 REPEXH02 strain PNUSAE043864

Strain PNUSAE043864 is a clinical isolate linked to the two multistate outbreaks which occurred in 2018 and 2019 (Waltenburg et al., 2021). The genome of strain PNUSAE043864 contains one chromosome (5,450,859 bp) and a large virulence plasmid pO157 (92,750 bp) (Table 1). PHASTER analysis revealed a total of 22 prophage and prophage-like elements on the chromosome, of which, 13 were complete, including a Stx2a-prophage (Table 2; Supplementary Table S1). Clade lineage analyses revealed that strain PNUSAE043864 belonged to clade 2, like strain TW14588, a lettuce isolate linked to the 2006 Taco John’s restaurants-associated outbreak of EcO157 infection in Iowa and Minnesota, and the REPEHX02 strain 2019C-3201 linked to multiple outbreaks from 2016 to 2019 in the U.S. (Table 1).

**Figure 2:**
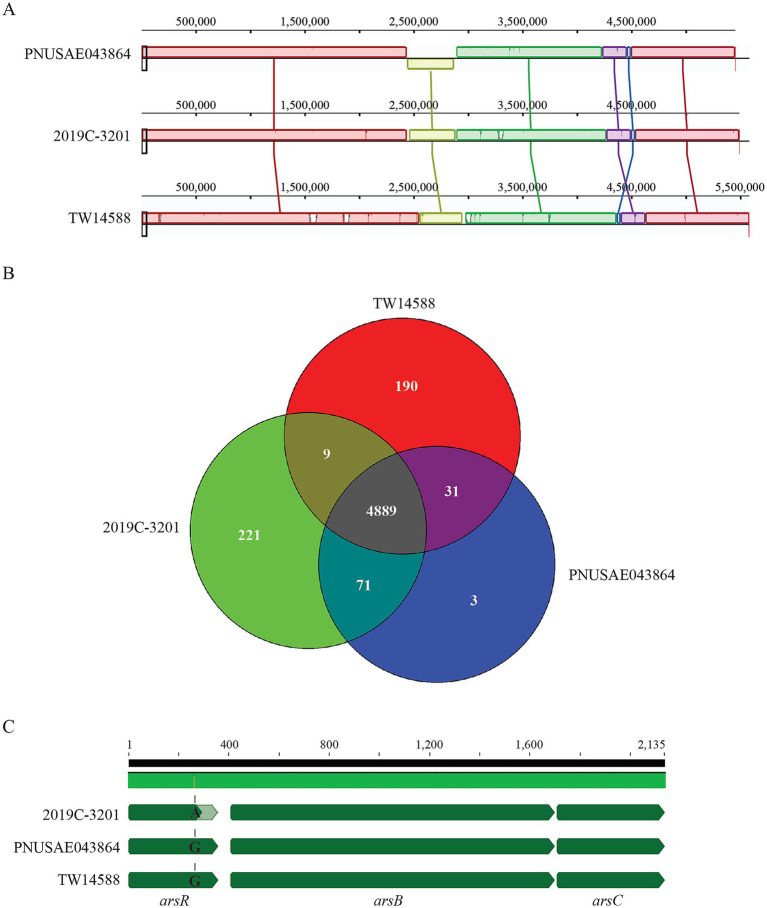
Comparative genomic analyses of the REPEXH02 strain PNUSAE043864. **(A)** Mauve alignment of chromosomes of strain PNUSAE043864 and the other two clade 2 strains, 2019C-3201 and TW14588. The numbers indicate the aligned base pair position. Both chromosomes of strains 2019C-3201 and TW14588 were re-oriented to the same start position as the chromosome of strain PNUSAE043864 prior to the analysis. The alignment was performed in Geneious Primer^®^2024.0.5 using Mauve Genome with progressive Mauve algorithm as described previously (Darling et al., 2004) with automatically calculating seed weight and the minimum LCB score. **(B)** Venn Diagram of shared and strain specific CDSs among the three genomes. Numbers of shared and strain specific orthologs were calculated in EDGAR 3.2 using default parameters. **(C)** Sequence analysis of the gene encoding ArsR, the As (III)-sensing metalloregulatory transcriptional repressor. The amber mutation present in strain 2019C-3201 (TGG to T**A**G) was not detected in *arsR* genes of strains PNUSAE043864 and TW14588.

Alignment of the chromosomes of the three strains revealed a large inversion (~ 433 Kb) in strain PNUSAE043864 (Figure 2A). The corresponding region in strain 2019C-3201 spanned the chromosomal positions from 1,608,107 to 2,041,794, containing a partial genome of prophage14, a complete genome of the prophage 15, and a partial genome of prophage 16. In strain TW14588, the corresponding region spanned the chromosomal positions from 4,595,581 to 4,986,609, containing a partial genome of prophage 17, a complete genome of prophage 18, and a partial genome of prophage 19. Comparative genomic analysis revealed that strain PNUSAE043864 shared more common genes with strain 2019C-3201 than with strain TW14588 (Figure 2B). Genes that were present in strain 2019C-3201 but not in strain PNUSAE043864 were mainly located on the secondary plasmids, 2019C-3201_p1 and 2019C-3201_p2, and several prophage genomes. Of the 34 genes that had no homologs in strain 2019C-3201, most of them were scattered across the chromosome, either encoding a hypothetical protein or a function related to MGEs. Other functions encoded by the PNUSAE043864 specific genes included heavy metal resistance, oxidative stress, virulence, and small toxic proteins (Supplementary Table S3). Specifically, the amber mutation identified in gene encoding As (III)-sensing metalloregulatory transcriptional repressor ArsR in strain 2019C-3201(TA*G) was not present in strains PNUSAE043864 and TW14588 (Figure 2C). Genes that were present in strain TW14588 but not in strain PNUSAE043864 were mainly found in prophage genomes, including the Stx1a-prophage and the Stx2a-prophage integrated next to tRNA gene *argW* (Table 2). Most of the functions encoded by TW14588 specific genes were phage related. Others included transcriptional regulators, adherence factors, and TTSS effectors.

The Stx2a-prophage in strain PNUSAE043864 was nearly identical to the Stx2a prophage in strain 2019C-3201 (97.9% identity) and exhibited higher sequence similarity with the Stx2a prophage inserted within the *wbrA* gene (94.7% Identity) in strain TW14588 than the Stx2a-prophage inserted next to *argW* gene (66.9% Identity). The Stx1a prophage in strain TW14588 was inserted at the same chromosome site as the ones utilized by the Stx1a-prophage in strain EDL933 (Table 2).

### 3.3 Biofilm formation on abiotic surfaces

**Figure 3:**
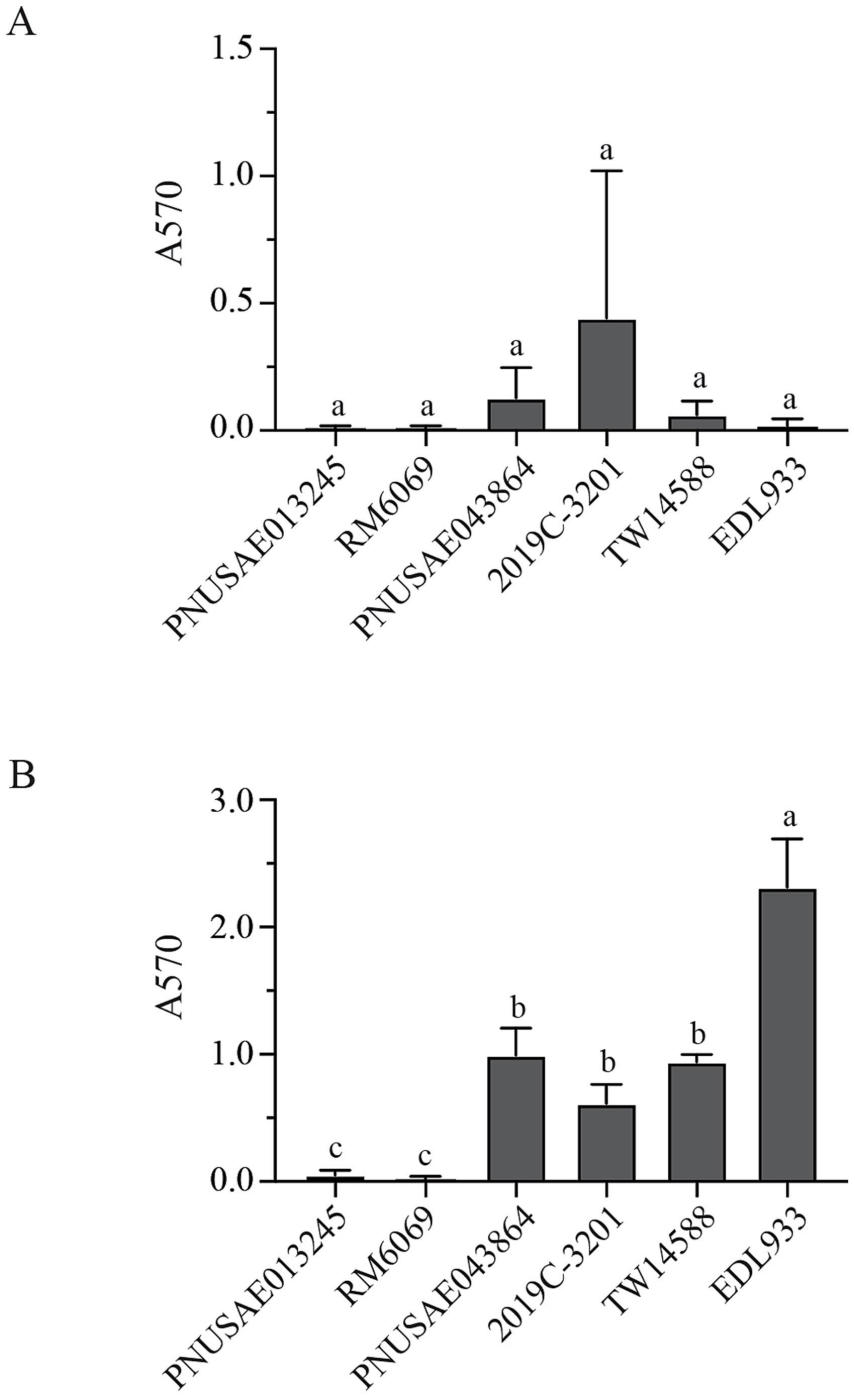
Biofilm formation by *E. coli* O157:H7 strain on glass surfaces. Quantitative analyses of biofilms under a static growth condition for 48 h **(A)** and 120 h **(B)**. The attached biomass on each tube was stained by crystal violet and quantified by the absorbance at 570 nm as detailed in Material and Methods. Each data set represents the mean and SD of three biological replicates. Differences that are statistically significant (One-way ANOVA followed by a Tukey’s multiple comparisons test, adjust *p* < 0.05) are indicated by the different letters.

Biofilm formation by EcO157 strains was evaluated quantitatively. Following 48 h incubation, a small amount of surface-associated biomass was detected for most of the strains examined, however, quantitative analyses revealed there were no significant differences among the strains examined (Figure 3A). When the incubation time increased to 120 h, a great strain variation in biofilm formation was observed (Figure 3B). Strain EDL933 produced the greatest amount of biofilm, followed by the three clade 2 strains. All three strains in clade 2 including the REPEXH02 strains PNUSAE043864 and 2019C-3201 produced greater amounts of biofilms than any of the two clade 8 strains, including the REPEXH01 strain PNUSAE013245 (One-way ANOVA, adjust *p* < 0.05). Consistent with a positive role of curli fimbriae in EcO157 biofilm formation on the glass surfaces (Carter et al., 2016), strains EDL933 and all three clade 2 strains were curli-expressing whereas both clade 8 strains were curli-deficient under the conditions used to test biofilm formation in this study.

### 3.4 Attachment to lettuce surfaces

**Figure 4:**
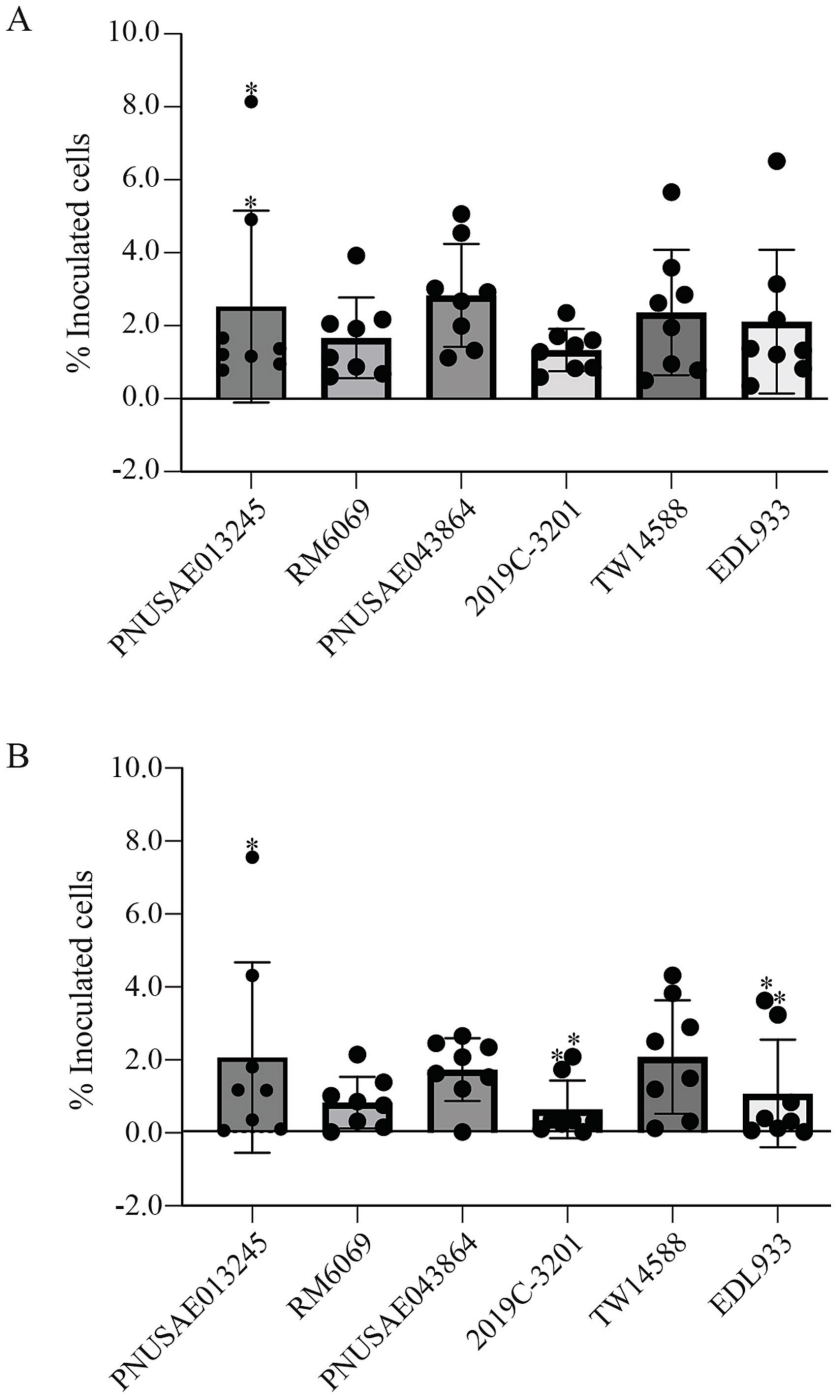
Attachment of *E. coli* O157:H7 to romaine lettuce surfaces. Quantitative analyses of loosely **(A)** and tightly **(B)** attached EcO157 cells on the lettuce leaves following spot inoculation as detailed in Material and Methods session. The data represent the average of eight biological replicates expressed as the percent of the corresponding initial inoculum. The data points marked with a “*” were outliners identified using ROUT method in Identify Outliers (Prism 10 version 10.2.3) when the aggressive value Q was at the default value (1%).

The capability of each EcO157 strain in adhesion to romaine lettuce surfaces was assessed by a spot inoculation method for loosely and tightly attached pathogen populations at the room temperature. For the six strains tested, the average of the loosely attached cells was between 1.3 and 2.8 percent of the inoculated cells (Figure 4A). The greatest amount of loosely attached cells was detected for the REPEXH02 strain PNUSAE043864, followed by the REPEXH01 strain PNUSAE013245, although the differences among the strains examined were not statistically significant (One-way ANOVA, adjusted *p* > 0.05). Furthermore, there were large variations in the attached cell population among the biological replicates of the same strain. For example, the percent loosely attached cells of the two outliers identified for the REPEXH01 strain PNUSAE013245 was 6.8- and 4.1-fold of the average (Figure 4A). The average of the tightly attached cells was between 0.7 and 2.1 percent of the inoculated cells (Figure 4B). The greatest amount of the tightly attached cells was detected for REPEXH01 strain PNUSAE013245 and strain TW14588, followed by the REPEXH02 strain PNUSAE043864, although the differences were not statistically significant (One-way ANOVA, adjust *p* > 0.05). More outliers were identified in the tightly attached cells compared with the loosely attached cells, including one for the REPEXH01 strain PNUSAE013245, two for the REPEXH02 strain 2019C-3201, and two for strain EDL933 (Figure 4B).

### 3.5 Population dynamics of *Escherichia coli* O157:H7 culturable cells in river water

**Figure 5:**
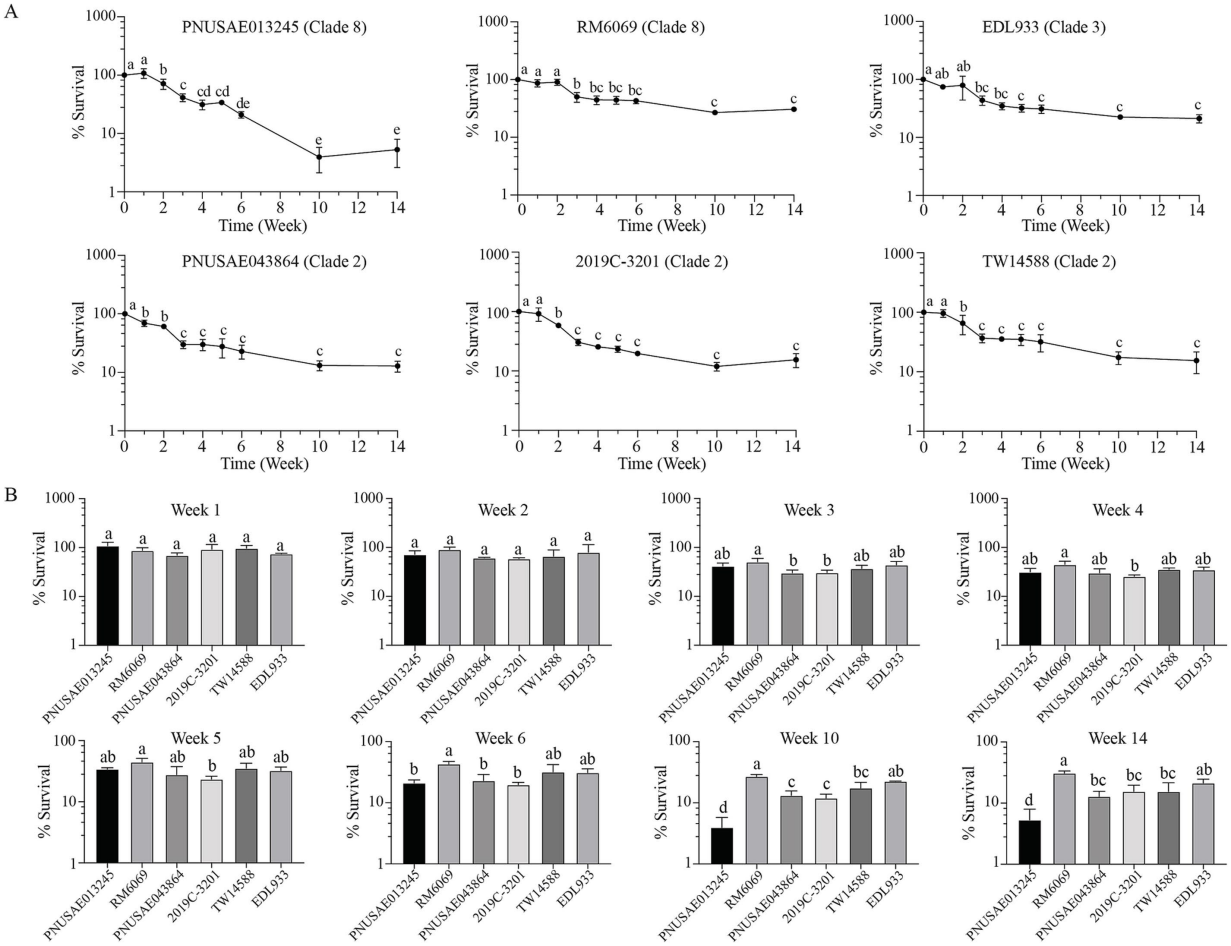
Population dynamics of *E. coli* O157:H7 culturable cells in river water. Survival of EcO157 in river water during incubation at 15°C for 14 weeks **(A)** and comparative analyses of the EcO157 survival in river water among strains belonging to different clades **(B)**. The population dynamics of EcO157 culturable cells were quantified by plate count for six strains inoculated in river water and incubated at 15°C for 14 weeks. Data represent the average percentage of EcO157 cells recovered on LB plates from three independent experiments. The error bars represent the standard deviation. Different letters represent the differences that are statistically significant determined by One-way ANOVA followed by a Tukey’s multiple comparisons test (adjust *p* < 0.05).

The population of EcO157 culturable cells in river water was examined by plate count during a 14-week cold incubation (15°C). Distinct survival trends were observed for the two clade 8 strains. For REPEXH01 strain PNUSAE013245, a significant reduction in culturable cells was detected following the first 2 weeks of incubation (Figure 5A). The culturable population continued to decease to 41.4, 21.0, and 3.9% of the total inoculated cells following 3-, 6-, and 10-weeks of incubation, respectively, and then remained stable following an additional four-week of incubation. A significant reduction in the culturable population of strain RM6069 was detected at week 3 post inoculation. The average culturable population was 42.9, 26.8, and 30.7% of the inoculated RM6069 cells following six, 10, and 14 weeks of incubation, respectively, which were significantly greater than the corresponding ones of the REPEXH01 strain PNUSAE013245 (One-way ANOVA adjust *p* < 0.05) (Figure 5B). Strain EDL933 exhibited a similar population dynamic as the 2006 spinach-associated outbreak strain RM6069 (Figure 5A, Clade 3). A significant decrease in the culturable population of EDL933 was detected following 3 weeks of cold incubation, and then followed by a slow reduction in the total culturable population through the entire incubation period although the differences were not statistically significant. The percent survivals for strain EDL933 at the week 10 and 14 post inoculation were all comparable with that of the strain RM6069 and significantly greater than that of the REPEXH01 strain PNUSAE013245 (Figure 5B).

Unlike the two clade 8 strains, the three strains within the clade 2 exhibited a similar survival trend in the river water. A significant decrease in the total culturable population was observed following 1 week of incubation for REPEXH02 strain PNUSAE043864 and 2 weeks of incubation for strains 2019C-3201 and TW14588. Following 3 weeks of incubation, the total culturable population decreased significantly for all three clade 2 strains, and then decayed slowly but not significantly till the end of incubation (Week 14). The average percent of survival on week 14 post inoculation was 12.8, 15.5, and 15.4 for strains PNUSAE043864, 2019C-3201, and TW14588, respectively (Figure 5A), which were all significantly lower than that of strain RM6069 but greater than that of the REPEXH01 strain PNUSAE013245 (Figure 5B).

### 3.6 Abundance of *Escherichia coli* O157:H7 persister cells in river water

**Figure 6:**
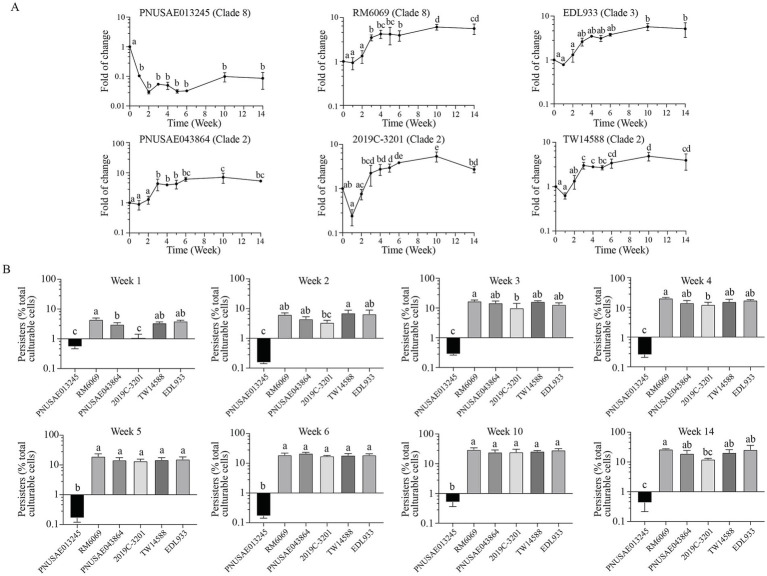
Abundance of *E. coli* O157:H7 persister cells in river water. Changes in EcO157 persister population in river water during incubation at 15°C for 14 weeks **(A)** and comparative analyses of the EcO157 persister population in river water among strains belonging to different clades **(B)**. The persister cells were determined for each sample including the inoculum of each strain by plate counts following the ciprofloxacin treatment. Therefore, the persister population was expressed as the percentage of total culturable cells (total CFUs). The data represent the mean and SD of survivals following a 24 h ciprofloxacin treatment from three biological replicates. Differences that are statistically significant (One-way ANOVA followed by a Tukey’s multiple comparisons test, adjust *p* < 0.05) are indicated by different letters.

Consistent with the distinct population dynamics of cultural cells between the two clade 8 strains, the dynamics of the persister populations differed greatly during the 14-week incubation (Figure 6A, Clade 8). For the REPEXH01 strain PNUSAE013245, the population size of persister cells decreased nearly 10-fold following the first week incubation and then remained in the range of 0.2–0.6% of total culturable cells over the period of 14 weeks (Figure 6B). In contrast, the persister population of the 2006 spinach-associated outbreak strain RM6069 started to increase significantly following the first three-week incubation and continued to increase significantly at week 10 post inoculation (Figure 6, RM6069). At the end of incubation, the persister population of strain RM6069 reached up to 26.6% of total culturable cells, which was more than 60-fold greater than the persister population of strain PNUSAE013245 although the fraction of persister cells in the initial inoculum was comparable for both strains (5.3% for strain PNUSAE013245 and 5.0% for RM6069). Unlike the clade 8 strains, the clade 2 strains exhibited a similar trend in production of persister cells during the 14-week incubation period. The population size of persister cells remained comparable with that in the initial inoculum (3.6–5.5%) during the first 2 weeks but started to increase at the week three post inoculation. For all three strains, the greatest persister population size was detected following 10 weeks of incubation, which was 24.1-, 24.4-, and 26.0% of the total culturable cells for strain PNUSAE043864, 2019C-3201, and TW14588, respectively (Figure 6B, Clade 2). A similar trend was detected for strain EDL933 (Clade 3). The largest persister population of EDL933 was detected during week 10 post inoculation which was nearly 6-fold of the persister fraction in the initial inoculum (5.0%).

Comparison of the dynamics of the persister populations among the EcO157 strains belonging to different clades did not reveal any clade-associated traits (Figure 6B). The persister population in the initial inoculum was at a similar level for all six strains examined, within a range of 3–5% of total culturable cells. Following the first week incubation, the persister populations of the REPEXH01 strain PNUSAE013245 and the REPEXH02 strain 2019C-3201 were significantly lower than that of any other strains. At 2 weeks of incubation, the persister population of strain PNUSAE013245 remained the lowest, while the persister population of strain 2019C-3201 started to increase and reached to a level comparable to all the other EcO157 strains except TW14588. The persister populations of strain PNUSAE013245 remained significantly lower than most of the other strains during the rest of incubation period (Figure 6B).

### 3.7 Abundance of *Escherichia coli* O157:H7 VBNC cells in river water

**Figure 7:**
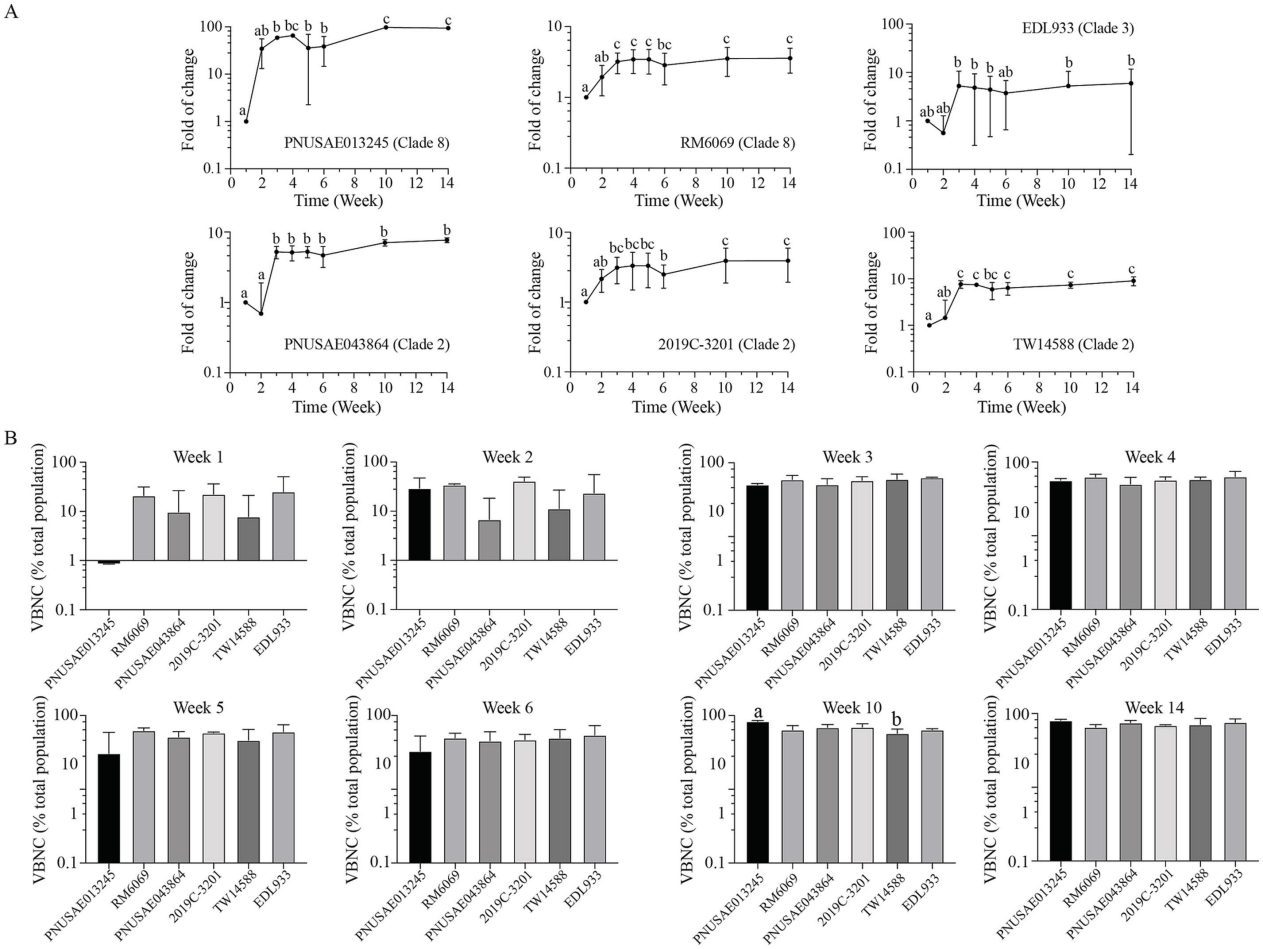
Abundance of *E. coli* O157:H7 VBNC cells in river water. Changes in EcO157 VBNC population in river water during incubation at 15°C for 14 weeks **(A)** and comparative analyses of the EcO157 VBNC population in river water among strains belonging to different clades **(B)**. The VBNC population was determined by calculating the difference between the total population determined by vPCR and the culturable population determined by plate counts and expressed as percentage of total population. The data represent the mean and SD of the VBNC population determined from three biological replicates. Differences that are statistically significant (One-way ANOVA followed by a Tukey’s multiple comparisons test, adjust *p* < 0.05) are indicated by different letters.

The population of VBNC cells for each EcO157 strain produced during the cold incubation in river water was evaluated by calculating the percent VBNC cells in the total population, which was a sum of culturable cells and the VBNC cells. An increase in VBNC population was observed for all EcO157 strains at week three post inoculation when compared with the corresponding VBNC population at week one (Figure 7A). The increase was significant for all strains except EDL933. The VBNC population continued to increase when the incubation time increased and reached to the highest level at week 10 for strains PNUSAE013245 and 2019C-3201, which were 82.6 and 69.4% of the total population, respectively. The greatest VBNC population for other strains were detected at week 14, which were in a range of 65–75% of the corresponding total population (Figure 7B). Unlike formation of persister cells, there were no significant differences in VBNC populations among the six strains tested at most sampling times (Figure 7B).

## 4 Discussion

EcO157 has been linked to numerous large outbreaks of gastrointestinal illness for more than 30 years. Strains of EcO157 differ in both genetic makeup and virulence potential. Comparative genomic analyses of over 500 clinical EcO157 strains led to the identification of 96 SNPs that grouped EcO157 strains in nine clades, among which, strains belonging to clade 8 appeared to be associated with higher rates of hospitalization and the development of HUS (Manning et al., 2008). Furthermore, the clade 8 strains are likely carrying a Stx2c-prophage. Consistent with this report, the two clade 8 strains examined in this study carry a Stx2a-prophage that are highly similar (% Identity, 93.5) and a Stx2c-prophage that are nearly identical (% Identity, 99.9). Both strains appeared to be hypervirulent when considering the rates of hospitalization and the number of HUS cases. During the 2006 spinach-associated outbreak, a total 225 people from 27 states were infected, 116 were hospitalized, 39 had HUS, and five died (Sharapov et al., 2016); During the 2018 lettuce-associated outbreak, a total of 240 people from 37 states were infected, 104 were hospitalized, 28 had HUS, and five died (Bottichio et al., 2020). Although the two strains were isolated over one decade apart and the environmental source of contamination was traced to California and Arizona, respectively, these two strains were genotypically highly related when their core genomes were compared. Both strains were placed in the SNP cluster PDS000181369 using NCBI Pathogen Detection pipeline (Table 1). As of September 5, 2024, a total of 3,960 EcO157 strains isolated from Europe, North America, and Asia, were placed in this SNP cluster. Among the 3,873 strains documented with isolation sources, 588 (15.2%) were environmental strains isolated from diverse samples including beef products, leafy greens, cattle, wildlife, animal waste, water, sediment, soil, and air dust. Interestingly, 78% environmental strains within this SNP cluster were isolated from California during 1994–2023, implying the persistence of EcO157 in leafy greens-growing environments.

Although the two clade 8 strains examined differed only in 64 loci based on the cgMLST analysis (Pathogen Detection), they belonged to different clusters when wgMLST was used for genotyping (EnteroBase, wgMLST, 3,393 for EC4115 and 68,417 for strain PNUSAE013245). Compared with the 2006 spinach-associated outbreak strain EC4115, the notable genomic features for the REPEXH01 strain PNUSAE013245 include the acquisition of a large GI carrying multiple antibiotic resistance (AR) genes and heavy metal resistance genes. The AR genes on this GI include *sul1*, *qacE*, *aadA1*, and *dfrA1* that are often found on integrons, implying a potential to spread these AR genes to other bacterial strains. This difference in the accessory genes repertoire between the two clade 8 strains demonstrated the rapid divergence driven by HGT among the EcO157 strains that are genotypically highly related. Acquisition of this large GI in the 2018 romaine lettuce-associated outbreak strains likely conferred the pathogen a fitness trait and contributed to the persistence and emergence of the REPEXH01 strains.

The REPEXH02 strain PNUSAE043864 belonged to the SNP cluster PDS000035073.188 that contains a total of 561 isolates as of September 5, 2024, including the REPEXH02 strain 2019C-3201 described previously (Chen et al., 2023). Most strains (459, ~ 82%) within this cluster are clinical. Although the REPEXH02 strains linked outbreaks were traced to 2016–2019, two clinical strains within this SNP cluster were isolated in Canada as early as 2011 (Biosample numbers, SAMN09738498 and SAMN09738483) and differed from the REPEXH02 strain PNUSAE043864 in less than 30 SNPs based on the cgMLST analyses. Of the 102 environmental strains within this SNP cluster, 83 were isolated from environmental samples collected in California during 2013–2021. The two water strains, RM19258 and RM19262, isolated by our research group in 2016, were placed into two branches within the SNP cluster PDS000035073.188. Strain RM19258 was grouped with the clinical strain PNUSAE043855 that was reported to PulseNet in December 2019, while strain RM19262 was grouped with the clinical strains reported to PulseNet in December 2016. Examining accessory genomes of the EcO157 strains linked to different outbreaks may reveal bacterial factors contributing to the reoccurrence of the REPEXH02 strains.

While the two REPEXH02 strains examined in this study share a highly conserved core genome (< 10 SNPs by cgMLST), they differed considerably in the content of plasmids and prophages. For example, strain PNUSAE043864 does not harbor any of the two secondary plasmids carried by the strain 2019C-3201. Furthermore, the amber mutation identified in gene encoding As (III)-sensing metalloregulatory transcriptional repressor ArsR in strain 2019C-3201(TA*G) was not present in strain PNUSAE043864. BLASTn search of nr Database in GenBank identified a sediment strain, 2018C-5284a, isolated in November 2018 in California, carries the same mutation in the *arsR* as in strain 2019C-3201. The *arsR* encodes a transcriptional repressor that binds to the promoter region of the *ars* operon. Upon interaction with arsenite, the ArsR repressor dissociates from the promoter region thus initiating the transcription of *ars* genes (Busenlehner et al., 2003). Therefore, disruption of ArsR in strain 2019C-3201 may lead to an increased expression of other *ars* genes. The *ars* operons examined in this study are composed of three genes, *arsRBC*, and located on the chromosomes. Deletion of chromosome-borne *arsRBC* in *E. coli* led to increased sensitivity to arsenite, antimonite, and arsenate; when the chromosome-borne *arsRBC* genes were expressed from a multicopy plasmid, they conferred *E. coli* cells a moderate-level of resistance to arsenite and antimonite (Carlin et al., 1995). Interestingly, a larger *ars* operon, *arsRDABC*, were detected in some STEC strains including several environmental strains isolated in the Salinas Valley. The *arsRDABC* operon was initially discovered on the *E. coli* plasmid R773 and has been associated with high-level arsenic resistances (Hedges and Baumberg, 1973; Ben Fekih et al., 2018). Additional studies are required to understand the functions of two *ars* operons in STEC and to examine if the mutation in the *arsR* gene would lead to the increased arsenic resistance in STEC.

The three clade 2 strains including the two REPEXH02 strains appear to be better biofilm producers than any of the clade 8 strains including the REPEXH01 strain when tested on the glass surfaces. This biofilm-producing proficiency appeared to be correlated with the curli fimbriae since, under the conditions tested for biofilms, curli fimbriae were detected for the three clade 2 strains and the control strain EDL933, but not for any of the clade 8 strains. This observation is consistent with our previous report on the roles of curli in surface attachment and biofilm formation that curli fimbriae enhanced biofilms of EcO157 on glass surfaces over 20-fold averagely (Carter et al., 2016). However, unlike our previous report that curli fimbriae significantly enhanced initial attachment of EcO157 to spinach leaves, there were no significant differences in the attachment of EcO157 to the lettuce surfaces among the six strains examined in this study. This discrepancy can be explained by the differences in the attachment assay systems. First, in our previous study that focused on illustrating the roles of curli fimbriae in attachment to various biotic and abiotic surfaces, the inoculation cultures were prepared under a condition that ensured the expression of curli fimbriae (Carter et al., 2016). Second, unlike the dip-inoculation method used in our previous study, the spot-inoculation method was used in this study to mimic a scenario of leafy greens contamination that could happen in preharvest fields, such as the deposition of fecal-droppings onto plant leaves. The 1-h incubation at 25°C used in the lettuce attachment assay might not fully facilitate the expression of curli fimbriae in EcO157. Following the surface washes, the cells released from the inoculated lettuce disks by vortexing were defined as “loosely attached” and by sonicating were defined as “tightly attached.” All six strains were comparable in the amounts of both “loosely” attached and “tightly” attached cells, suggesting that all EcO157 strains examined in this study share comparable proficiency in adhesion to lettuce surface. Besides genes encoding curli fimbriae, all EcO157 strains carried genes encoding *E. coli* common pilus (ECP), hemorrhagic *E. coli* pilus (HCP), type 1 fimbriae, and several protein adhesins such as Cah, EaeH, EhaA, and EhaG. Mutations in several adhesins genes in the EcO157 REPEXH02 strains reported previously including *bigA*, *fdeC*, and *yeeJ* (Cherry, 2022), were detected in the two REPEXH02 strains examined in this study but not in other strains, implying a minimal impact of these mutations on the attachment of EcO157 to lettuce leaves. The large variations in lettuce adhesion among the biological replicates of the same strain were likely attributed to the variations in the topographical and physiochemical properties of the lettuce surfaces. Attachment of *Salmonella* to different lettuce leaf regions is highly variable and impacted by the lettuce age (Kroupitski et al., 2011). Retention of *E. coli* on the spinach leaf surfaces was reported to be impacted by leaf vein density, available nutrients, leaf age, and leaf axis (Doan et al., 2020). Therefore, interactions between the EcO157 cells and the leave surfaces are complex and likely impacted by physical, chemical, and biological factors including bacterial surface properties, leafy surface properties, and the environmental conditions. Failure to remove EcO157 from the inoculated lettuce leaves by sonication highlights the challenges in the development of effective intervention strategies to ensure microbial safety of leafy greens.

Although bacterial persister cells were first discovered due to their increased antibiotic resistances, persisters display high tolerance to other stresses such as sanitizers, starvation, and UV irritation (Harms et al., 2016; Van den Bergh et al., 2017). Therefore, formation of persisters by foodborne pathogens would enhance their environment persistence and pose significant challenges to food safety. Formation of EcO157 persister cells were reported in field water, spinach leaf wash, and on lettuce plants grown in laboratory (Thao et al., 2019; Munther et al., 2020). In both studies, a high level of persister cells was detected when EcO157 displayed a slow growth or within a declining population. Consistent with our previous report, a high level of EcO157 persisters (25–30% of inoculated population) was detected for five out of six EcO157 strains examined (Figure 6). The level of persisters in both REPEXH02 strains was similar to that in non-REP strains, while the level of persisters in the REPEXH01 strain PNUSAE013245 during the late stage of incubation were significantly lower than that in any other EcO157 strains. Variation in formation of persisters were reported for different species and strains (Van den Bergh et al., 2017). Persister cells are produced stochastically and can be induced in response to environmental cues, such as the conditions that promote the production of (p)ppGpp, or signals that activate stress responses (Harms et al., 2016). Therefore, mutations in genes involved in the cellular signaling upstream of the persister formation may lead to defect in producing persister cells. For example, mutants unable to produce (p)ppGpp, exhibited reduced levels of persisters in *Pseudomona aeruginosa* (Viducic et al., 2006). Mutants defective in amino acid biosynthesis or metabolism were reported to display altered persister formation in *E. coli* (Girgis et al., 2012). Additional studies are required to understand the mechanism underlying the persister formation in REP strains, and how the persister sub-population contributes to persistence and re-emergence of REP strains.

Like persisters, VBNC cells are another form of physiological variants and can be induced by various chemicals and environmental factors (Oliver, 2010; Liu et al., 2023). Unlike the formation of persister cells, the VBNC population for all six strains examined in this study were comparable after 3 weeks of incubation in river water. Interestingly, the VBNC population of the REPEXH01 strain PNUSAE013245, which was defective in the formation of persisters, was larger than any of the other strains at the late stage of incubation (Figure 7). At the end of the 14-week incubation, the VBNC population of EcO157 strains were in the range of 65–80% of the total population, implying VBNC cells may be a reservoir of pathogen cells in the natural environment. Presence of VBNC cells of human pathogens in the food production environments is a big concern considering their potential to resuscitate to actively growing and infectious cells. VBNC cells of EcO157 were reported in the phyllosphere of lettuce and in the process water treated by peroxyacetic acid, chlorine dioxide, and chlorine, the common disinfectants used in the fresh-cut industry (Dinu and Bach, 2011; Truchado et al., 2021; Truchado et al., 2023). Resuscitation of *L. monocytogenes* VBNC cells on shredded lettuce was observed during storage (Truchado et al., 2023). Resuscitation of *L. monocytogenes’* VBNC cells was also reported in the nematode intestinal tract, where resuscitated cells were infectious (Highmore et al., 2018). Information about resuscitation of EcO157 VBNC cells is limited. Research on conditions promoting resuscitation of EcO157 VBNC cells induced in the leafy greens pre- and postharvest environments are desired since such knowledge will be fundamental in advancing safety of leafy greens.

The persistence of STEC in leafy greens production environments poses significant challenges in the efforts to reduce STEC contamination and outbreaks. Recent identification of EcO157 REP strains emphasizes the need to understand the underlying mechanisms of reoccurrence, emergence, and persistence. EcO157 has adopted various mechanisms to maximize their survival in natural environment. Genetic diversification through mutations and HGT is a known key factor driving virulence evolution in EcO157. Although the REPEXH01 strain PNUSAE013245 linked outbreaks were traced back to 2017, a food strain belonging to the same SNP cluster (Table 1) was isolated as early as 1993. It will be informative to reveal temporal genetic changes among the strains belonging to the SNP cluster PDS000181369, especially those linked to the 2006-spinach associated outbreak and to the 2018 romaine lettuce-associated outbreak. Besides genetic diversification, phenotypic diversification has been reported in EcO157 populations for improved fitness (Carter et al., 2011). Unlike mutation-mediated phenotypic diversification, a bet-hedging strategy was suggested for the production of persisters and VBNC cells (Lennon and Jones, 2011), the two highly stress-tolerant physiological states, representing a continuum between actively growing and dead cells, with VBNC cells being in a deeper state of dormancy than persister cells (Ayrapetyan et al., 2015; Ayrapetyan et al., 2018). This dormancy allows pathogen cells to survive under stressful conditions and to revive once the condition becomes permissive. Persisters and VBNC cells share certain physiological properties and the molecular mechanisms underlying the induction of dormancy, however, persisters appear to exit dormancy and be resuscitated more rapidly than the VBNC cells. Persisters and VBNC cells of EcO157 represent two subpopulations with common and distinct properties that are needed to be considered in risk assessment and in the development of control strategies.

## Data Availability

The datasets presented in this study can be found in online repositories. The names of the repository/repositories and accession number(s) can be found in the article/Table 1.

## Footnotes

^1^ https://www.cdc.gov/ncezid/dfwed/outbreak-response/rep-strains/repexh01.html#print

## Author disclaimer

This project used Material from the California Department of Public Health. The findings and conclusions in this article are those of the authors and do not necessarily represent the views or opinions of the California Department of Public Health or the California Health and Human Services Agency. This research used Material from the Center for Diseases Control and Prevention. The findings and conclusions in this report are those of the authors and do not necessarily represent the official position of the Centers for Disease Control and Prevention, the Department of Health and Human Services, or the United States government. Furthermore, the use of any product names, trade names, images, or commercial sources is for identification purposes only, and does not imply endorsement or government sanction by the U.S. Department of Health and Human Services.

## References

[ref1] Arndt D. Grant J. R. Marcu A. Sajed T. Pon A. Liang Y.. (2016). PHASTER: a better, faster version of the PHAST phage search tool. Nucleic Acids Res. 44, W16–W21. doi: 10.1093/nar/gkw387, PMID: 27141966 PMC4987931

[ref2] Ayrapetyan M. Williams T. C. Baxter R. Oliver J. D. (2015). Viable but Nonculturable and Persister cells coexist stochastically and are induced by human serum. Infect. Immun. 83, 4194–4203. doi: 10.1128/IAI.00404-15, PMID: 26283335 PMC4598401

[ref3] Ayrapetyan M. Williams T. Oliver J. D. (2018). Relationship between the viable but nonculturable state and antibiotic persister cells. J. Bacteriol. 200:e00249-18. doi: 10.1128/JB.00249-18 30082460 PMC6153661

[ref4] Balaban N. Q. Merrin J. Chait R. Kowalik L. Leibler S. (2004). Bacterial persistence as a phenotypic switch. Science 305, 1622–1625. doi: 10.1126/science.1099390 15308767

[ref5] Ben Fekih I. Zhang C. Li Y. P. Zhao Y. Alwathnani H. A. Saquib Q.. (2018). Distribution of arsenic resistance genes in prokaryotes. Front. Microbiol. 9:2473. doi: 10.3389/fmicb.2018.02473 30405552 PMC6205960

[ref6] Berger C. N. Sodha S. V. Shaw R. K. Griffin P. M. Pink D. Hand P.. (2010). Fresh fruit and vegetables as vehicles for the transmission of human pathogens. Environ. Microbiol. 12, 2385–2397. doi: 10.1111/j.1462-2920.2010.02297.x 20636374

[ref7] Bottichio L. Keaton A. Thomas D. Fulton T. Tiffany A. Frick A.. (2020). Shiga toxin-producing *Escherichia coli* infections associated with Romaine lettuce-United States, 2018. Clin. Infect. Dis. 71, e323–e330. doi: 10.1093/cid/ciz1182 31814028 PMC10982825

[ref8] Brandl M. T. (2006). Fitness of human enteric pathogens on plants and implications for food safety. Annu. Rev. Phytopathol. 44, 367–392. doi: 10.1146/annurev.phyto.44.070505.143359, PMID: 16704355

[ref9] Burland V. Shao Y. Perna N. T. Plunkett G. Sofia H. J. Blattner F. R. (1998). The complete DNA sequence and analysis of the large virulence plasmid of *Escherichia coli* O157:H7. Nucleic Acids Res. 26, 4196–4204. doi: 10.1093/nar/26.18.4196, PMID: 9722640 PMC147824

[ref1002] Busenlehner L. S. Pennella M. A. Giedroc D. P. (2003). The SmtB/ArsR family of metalloregulatory transcriptional repressors: Structural insights into prokaryotic metal resistance. FEMS Microbiol Rev. 27, 131–143. doi: 10.1016/S0168-6445(03)00054-8 12829264

[ref10] Carlin A. Shi W. Dey S. Rosen B. P. (1995). The *ars* operon of *Escherichia coli* confers arsenical and antimonial resistance. J. Bacteriol. 177, 981–986. doi: 10.1128/jb.177.4.981-986.1995, PMID: 7860609 PMC176692

[ref11] Carrascosa C. Raheem D. Ramos F. Saraiva A. Raposo A. (2021). Microbial biofilms in the food industry-a comprehensive review. Int. J. Environ. Res. Public Health 18:2014. doi: 10.3390/ijerph18042014, PMID: 33669645 PMC7922197

[ref12] Carter M. Q. Brandl M. T. Kudva I. T. Katani R. Moreau M. R. Kapur V. (2018). Conditional function of autoaggregative protein Cah and common *cah* mutations in Shiga toxin-producing *Escherichia coli*. Appl. Environ. Microbiol. 84:e01739-17. doi: 10.1128/AEM.01739-17, PMID: 29054868 PMC5734025

[ref13] Carter M. Q. Brandl M. T. Louie J. W. Kyle J. L. Carychao D. K. Cooley M. B.. (2011). Distinct acid resistance and survival fitness displayed by curli variants of enterohemorrhagic *Escherichia coli* O157:H7. Appl. Environ. Microbiol. 77, 3685–3695. doi: 10.1128/AEM.02315-10 21478320 PMC3127601

[ref14] Carter M. Q. Feng D. Li H. H. (2019). Curli fimbriae confer Shiga toxin-producing *Escherichia coli* a competitive trait in mixed biofilms. Food Microbiol. 82, 482–488. doi: 10.1016/j.fm.2019.03.024 31027809

[ref15] Carter M. Q. Louie J. W. Feng D. Zhong W. Brandl M. T. (2016). Curli fimbriae are conditionally required in *Escherichia coli* O157:H7 for initial attachment and biofilm formation. Food Microbiol. 57, 81–89. doi: 10.1016/j.fm.2016.01.006, PMID: 27052705

[ref16] Carter M. Q. Quinones B. He X. Pham A. Carychao D. Cooley M. B.. (2023). Genomic and phenotypic characterization of Shiga toxin-producing *Escherichia albertii* strains isolated from wild birds in a major agricultural region in California. Microorganisms 11:2803. doi: 10.3390/microorganisms11112803, PMID: 38004814 PMC10673567

[ref17] Chen J. C. Patel K. Smith P. A. Vidyaprakash E. Snyder C. Tagg K. A.. (2023). Reoccurring *Escherichia coli* O157:H7 strain linked to leafy greens-associated outbreaks, 2016-2019. Emerg. Infect. Dis. 29, 1895–1899. doi: 10.3201/eid2909.230069, PMID: 37610207 PMC10461648

[ref18] Cherry J. L. (2022). Recent genetic changes affecting Enterohemorrhagic *Escherichia coli* causing recurrent outbreaks. Microbiol. Spectr. 10:e0050122. doi: 10.1128/spectrum.00501-22, PMID: 35467376 PMC9241674

[ref19] Chitlapilly Dass S. Wang R. (2022). Biofilm through the looking glass: a microbial food safety perspective. Pathogens 11:346. doi: 10.3390/pathogens11030346, PMID: 35335670 PMC8954374

[ref20] Cooley M. B. Quinones B. Oryang D. Mandrell R. E. Gorski L. (2014). Prevalence of Shiga toxin producing *Escherichia coli*, *Salmonella enterica*, and *Listeria monocytogenes* at public access watershed sites in a California central coast agricultural region. Front. Cell. Infect. Microbiol. 4:30. doi: 10.3389/fcimb.2014.00030, PMID: 24624367 PMC3940966

[ref21] Darling A. C. Mau B. Blattner F. R. Perna N. T. (2004). Mauve: multiple alignment of conserved genomic sequence with rearrangements. Genome Res. 14, 1394–1403. doi: 10.1101/gr.2289704, PMID: 15231754 PMC442156

[ref22] Dinu L. D. Bach S. (2011). Induction of viable but nonculturable *Escherichia coli* O157:H7 in the phyllosphere of lettuce: a food safety risk factor. Appl. Environ. Microbiol. 77, 8295–8302. doi: 10.1128/AEM.05020-11, PMID: 21965401 PMC3233046

[ref23] Doan H. K. Antequera-Gomez M. L. Parikh A. N. Leveau J. H. J. (2020). Leaf surface topography contributes to the ability of *Escherichia coli* on leafy greens to resist removal by washing, escape disinfection with chlorine, and disperse through splash. Front. Microbiol. 11:1485. doi: 10.3389/fmicb.2020.01485, PMID: 32765440 PMC7380079

[ref24] Dorr T. Lewis K. Vulic M. (2009). SOS response induces persistence to fluoroquinolones in *Escherichia coli*. PLoS Genet. 5:e1000760. doi: 10.1371/journal.pgen.1000760, PMID: 20011100 PMC2780357

[ref25] Eppinger M. Mammel M. K. Leclerc J. E. Ravel J. Cebula T. A. (2011). Genomic anatomy of *Escherichia coli* O157:H7 outbreaks. Proc. Natl. Acad. Sci. USA 108, 20142–20147. doi: 10.1073/pnas.1107176108, PMID: 22135463 PMC3250189

[ref26] Girgis H. S. Harris K. Tavazoie S. (2012). Large mutational target size for rapid emergence of bacterial persistence. Proc. Natl. Acad. Sci. USA 109, 12740–12745. doi: 10.1073/pnas.1205124109, PMID: 22802628 PMC3411964

[ref27] Griffin P. M. Tauxe R. V. (1991). The epidemiology of infections caused by *Escherichia coli* O157:H7, other enterohemorrhagic *E. Coli*, and the associated hemolytic uremic syndrome. Epidemiol. Rev. 13, 60–98. doi: 10.1093/oxfordjournals.epirev.a036079 1765120

[ref28] Harms A. Maisonneuve E. Gerdes K. (2016). Mechanisms of bacterial persistence during stress and antibiotic exposure. Science 354:aaf4268. doi: 10.1126/science.aaf4268 27980159

[ref29] Hedges R. W. Baumberg S. (1973). Resistance to arsenic compounds conferred by a plasmid transmissible between strains of *Escherichia coli*. J. Bacteriol. 115, 459–460. doi: 10.1128/jb.115.1.459-460.1973 4577750 PMC246262

[ref30] Heiman K. E. Mody R. K. Johnson S. D. Griffin P. M. Gould L. H. (2015). *Escherichia coli* O157 outbreaks in the United States, 2003-2012. Emerg. Infect. Dis. 21, 1293–1301. doi: 10.3201/eid2108.141364, PMID: 26197993 PMC4517704

[ref31] Highmore C. J. Warner J. C. Rothwell S. D. Wilks S. A. Keevil C. W. (2018). Viable-but-nonculturable *listeria monocytogenes* and *Salmonella enterica* Serovar Thompson induced by chlorine stress remain infectious. mBio 9:e00540-18. doi: 10.1128/mBio.00540-18 29666286 PMC5904417

[ref32] Jay M. T. Cooley M. Carychao D. Wiscomb G. W. Sweitzer R. A. Crawford-Miksza L.. (2007). *Escherichia coli* O157:H7 in feral swine near spinach fields and cattle, Central California coast. Emerg. Infect. Dis. 13, 1908–1911. doi: 10.3201/eid1312.070763, PMID: 18258044 PMC2876768

[ref33] Johnson P. J. Levin B. R. (2013). Pharmacodynamics, population dynamics, and the evolution of persistence in *Staphylococcus aureus*. PLoS Genet. 9:e1003123. doi: 10.1371/journal.pgen.1003123, PMID: 23300474 PMC3536638

[ref34] Kilonzo C. Li X. Vivas E. J. Jay-Russell M. T. Fernandez K. L. Atwill E. R. (2013). Fecal shedding of zoonotic food-borne pathogens by wild rodents in a major agricultural region of the Central California coast. Appl. Environ. Microbiol. 79, 6337–6344. doi: 10.1128/AEM.01503-13, PMID: 23934490 PMC3811224

[ref1003] Kint C. I. Verstraeten N. Fauvart M. Michiels J. (2012). New-found fundamentals of bacterial persistence. Trends Microbiol. 20, 577–585. doi: 10.1016/j.tim.2012.08.009, PMID: 22959615

[ref35] Kroupitski Y. Pinto R. Belausov E. Sela S. (2011). Distribution of *Salmonella typhimurium* in romaine lettuce leaves. Food Microbiol. 28, 990–997. doi: 10.1016/j.fm.2011.01.007, PMID: 21569943

[ref36] Lennon J. T. Jones S. E. (2011). Microbial seed banks: the ecological and evolutionary implications of dormancy. Nat. Rev. Microbiol. 9, 119–130. doi: 10.1038/nrmicro2504, PMID: 21233850

[ref37] Lewis K. (2010). Persister cells. Ann. Rev. Microbiol. 64, 357–372. doi: 10.1146/annurev.micro.112408.134306 20528688

[ref38] Lewis K. (2012). Persister cells: molecular mechanisms related to antibiotic tolerance. Handb. Exp. Pharmacol. 211, 121–133. doi: 10.1007/978-3-642-28951-4_8 23090599

[ref39] Li L. Mendis N. Trigui H. Oliver J. D. Faucher S. P. (2014). The importance of the viable but non-culturable state in human bacterial pathogens. Front. Microbiol. 5:258. doi: 10.3389/fmicb.2014.00258, PMID: 24917854 PMC4040921

[ref40] Liu J. Yang L. Kjellerup B. V. Xu Z. (2023). Viable but nonculturable (VBNC) state, an underestimated and controversial microbial survival strategy. Trends Microbiol. 31, 1013–1023. doi: 10.1016/j.tim.2023.04.009 37225640

[ref41] Luo H. Zhang C. T. Gao F. (2014). Ori-finder 2, an integrated tool to predict replication origins in the archaeal genomes. Front. Microbiol. 5:482. doi: 10.3389/fmicb.2014.00482 25309521 PMC4164010

[ref42] Manning S. D. Motiwala A. S. Springman A. C. Qi W. Lacher D. W. Ouellette L. M.. (2008). Variation in virulence among clades of *Escherichia coli* O157:H7 associated with disease outbreaks. Proc. Natl. Acad. Sci. USA 105, 4868–4873. doi: 10.1073/pnas.0710834105, PMID: 18332430 PMC2290780

[ref43] Marshall K. E. Hexemer A. Seelman S. L. Fatica M. K. Blessington T. Hajmeer M.. (2020). Lessons learned from a decade of investigations of Shiga toxin-producing *Escherichia coli* outbreaks linked to leafy greens, United States and Canada. Emerg. Infect. Dis. 26, 2319–2328. doi: 10.3201/eid2610.191418, PMID: 32946367 PMC7510726

[ref44] Munther D. S. Carter M. Q. Aldric C. V. Ivanek R. Brandl M. T. (2020). Formation of *Escherichia coli* O157:H7 Persister cells in the lettuce Phyllosphere and application of differential equation models to predict their prevalence on lettuce plants in the field. Appl. Environ. Microbiol. 86:e01602-19. doi: 10.1128/AEM.01602-19, PMID: 31704677 PMC6952222

[ref45] Navarro-Gonzalez N. Wright S. Aminabadi P. Gwinn A. Suslow T. V. Jay-Russell M. T. (2020). Carriage and subtypes of foodborne pathogens identified in wild birds residing near agricultural lands in California: a repeated cross-sectional study. Appl. Environ. Microbiol. 86:e01678-19. doi: 10.1128/AEM.01678-19, PMID: 31757824 PMC6974635

[ref46] Nguyen D. Joshi-Datar A. Lepine F. Bauerle E. Olakanmi O. Beer K.. (2011). Active starvation responses mediate antibiotic tolerance in biofilms and nutrient-limited bacteria. Science 334, 982–986. doi: 10.1126/science.1211037, PMID: 22096200 PMC4046891

[ref47] Oliver J. D. (2010). Recent findings on the viable but nonculturable state in pathogenic bacteria. FEMS Microbiol. Rev. 34, 415–425. doi: 10.1111/j.1574-6976.2009.00200.x, PMID: 20059548

[ref48] Perna N. T. Plunkett G. 3rd Burland V. Mau B. Glasner J. D. Rose D. J.. (2001). Genome sequence of enterohaemorrhagic *Escherichia coli* O157:H7. Nature 409, 529–533. doi: 10.1038/35054089 11206551

[ref49] Riordan J. T. Viswanath S. B. Manning S. D. Whittam T. S. (2008). Genetic differentiation of *Escherichia coli* O157:H7 clades associated with human disease by real-time PCR. J. Clin. Microbiol. 46, 2070–2073. doi: 10.1128/JCM.00203-08, PMID: 18400915 PMC2446876

[ref50] Ryu J. H. Beuchat L. R. (2005). Biofilm formation by *Escherichia coli* O157:H7 on stainless steel: effect of exopolysaccharide and Curli production on its resistance to chlorine. Appl. Environ. Microbiol. 71, 247–254. doi: 10.1128/AEM.71.1.247-254.2005 15640194 PMC544232

[ref51] Sharapov U. M. Wendel A. M. Davis J. P. Keene W. E. Farrar J. Sodha S.. (2016). Multistate outbreak of *Escherichia coli* O157:H7 infections associated with consumption of fresh spinach: United States, 2006. J. Food Prot. 79, 2024–2030. doi: 10.4315/0362-028X.JFP-15-556 28221950

[ref52] Strawn L. K. Grohn Y. T. Warchocki S. Worobo R. W. Bihn E. A. Wiedmann M. (2013). Risk factors associated with *Salmonella* and *Listeria monocytogenes* contamination of produce fields. Appl. Environ. Microbiol. 79, 7618–7627. doi: 10.1128/AEM.02831-13, PMID: 24077713 PMC3837806

[ref53] Tatusova T. DiCuccio M. Badretdin A. Chetvernin V. Nawrocki E. P. Zaslavsky L.. (2016). NCBI prokaryotic genome annotation pipeline. Nucleic Acids Res. 44, 6614–6624. doi: 10.1093/nar/gkw569, PMID: 27342282 PMC5001611

[ref54] Thao S. Brandl M. T. Carter M. Q. (2019). Enhanced formation of Shiga toxin-producing *Escherichia coli* persister variants in environments relevant to leafy greens production. Food Microbiol. 84:103241. doi: 10.1016/j.fm.2019.103241 31421768

[ref55] Truchado P. Gil M. I. Allende A. (2021). Peroxyacetic acid and chlorine dioxide unlike chlorine induce viable but non-culturable (VBNC) stage of listeria monocytogenes and *Escherichia coli* O157:H7 in wash water. Food Microbiol. 100:103866. doi: 10.1016/j.fm.2021.103866, PMID: 34416966

[ref56] Truchado P. Gomez-Galindo M. Gil M. I. Allende A. (2023). Cross-contamination of *Escherichia coli* O157:H7 and *Listeria monocytogenes* in the viable but non-culturable (VBNC) state during washing of leafy greens and the revival during shelf-life. Food Microbiol. 109:104155. doi: 10.1016/j.fm.2022.104155, PMID: 36309451

[ref57] Tuttle J. Gomez T. Doyle M. P. Wells J. G. Zhao T. Tauxe R. V.. (1999). Lessons from a large outbreak of *Escherichia coli* O157:H7 infections: insights into the infectious dose and method of widespread contamination of hamburger patties. Epidemiol. Infect. 122, 185–192. doi: 10.1017/s0950268898001976, PMID: 10355781 PMC2809605

[ref58] Van den Bergh B. Fauvart M. Michiels J. (2017). Formation, physiology, ecology, evolution and clinical importance of bacterial persisters. FEMS Microbiol. Rev. 41, 219–251. doi: 10.1093/femsre/fux001, PMID: 28333307

[ref59] Viducic D. Ono T. Murakami K. Susilowati H. Kayama S. Hirota K.. (2006). Functional analysis of *spoT*, *relA* and *dksA* genes on quinolone tolerance in *Pseudomonas aeruginosa* under nongrowing condition. Microbiol. Immunol. 50, 349–357. doi: 10.1111/j.1348-0421.2006.tb03793.x, PMID: 16625057

[ref60] Vogeleer P. Tremblay Y. D. Mafu A. A. Jacques M. Harel J. (2014). Life on the outside: role of biofilms in environmental persistence of Shiga-toxin producing *Escherichia coli*. Front. Microbiol. 5:317. doi: 10.3389/fmicb.2014.00317, PMID: 25071733 PMC4076661

[ref61] Waltenburg M. A. Schwensohn C. Madad A. Seelman S. L. Peralta V. Koske S. E.. (2021). Two multistate outbreaks of a reoccurring Shiga toxin-producing *Escherichia coli* strain associated with romaine lettuce: USA, 2018-2019. Epidemiol. Infect. 150:e16. doi: 10.1017/S0950268821002703, PMID: 35060456 PMC8796143

[ref62] Wang G. Doyle M. P. (1998). Survival of enterohemorrhagic *Escherichia coli* O157:H7 in water. J. Food Prot. 61, 662–667. doi: 10.4315/0362-028x-61.6.662 9709245

[ref63] Watnick P. Kolter R. (2000). Biofilm, city of microbes. J. Bacteriol. 182, 2675–2679. doi: 10.1128/JB.182.10.2675-2679.2000, PMID: 10781532 PMC101960

[ref64] Wei C. Zhao X. (2018). Induction of viable but Nonculturable *Escherichia coli* O157:H7 by low temperature and its resuscitation. Front. Microbiol. 9:2728. doi: 10.3389/fmicb.2018.02728, PMID: 30555428 PMC6282054

[ref65] Wiegand I. Hilpert K. Hancock R. E. (2008). Agar and broth dilution methods to determine the minimal inhibitory concentration (MIC) of antimicrobial substances. Nat. Protoc. 3, 163–175. doi: 10.1038/nprot.2007.521, PMID: 18274517

[ref66] Wu Y. Vulic M. Keren I. Lewis K. (2012). Role of oxidative stress in persister tolerance. Antimicrob. Agents Chemother. 56, 4922–4926. doi: 10.1128/AAC.00921-12, PMID: 22777047 PMC3421885

[ref67] Xu H. S. Roberts N. Singleton F. L. Attwell R. W. Grimes D. J. Colwell R. R. (1982). Survival and viability of nonculturable *Escherichia coli* and *Vibrio cholerae* in the estuarine and marine environment. Microb. Ecol. 8, 313–323. doi: 10.1007/BF02010671, PMID: 24226049

[ref68] Yan J. Bassler B. L. (2019). Surviving as a community: antibiotic tolerance and persistence in bacterial biofilms. Cell Host Microbe 26, 15–21. doi: 10.1016/j.chom.2019.06.002, PMID: 31295420 PMC6629468

[ref69] Yaron S. Romling U. (2014). Biofilm formation by enteric pathogens and its role in plant colonization and persistence. Microb. Biotechnol. 7, 496–516. doi: 10.1111/1751-7915.12186, PMID: 25351039 PMC4265070

[ref70] Zhang S. Ye C. Lin H. Lv L. Yu X. (2015). UV disinfection induces a VBNC state in *Escherichia coli* and *Pseudomonas aeruginosa*. Environ. Sci. Technol. 49, 1721–1728. doi: 10.1021/es505211e 25584685

[ref71] Zhao S. Zhang J. Li Z. Han Y. Kan B. (2021). Enumeration of viable non-culturable *Vibrio cholerae* using droplet digital PCR combined with Propidium Monoazide treatment. Front. Cell. Infect. Microbiol. 11:753078. doi: 10.3389/fcimb.2021.753078, PMID: 34796126 PMC8592976

